# Dietary Supplementation with Spray-Dried Porcine Plasma Attenuates Colon Inflammation in a Genetic Mouse Model of Inflammatory Bowel Disease

**DOI:** 10.3390/ijms21186760

**Published:** 2020-09-15

**Authors:** Lluïsa Miró, Concepció Amat, Cristina Rosell-Cardona, Joy M. Campbell, Javier Polo, Anna Pérez-Bosque, Miquel Moretó

**Affiliations:** 1 Dept. of Biochemistry and Physiology, Faculty of Pharmacy and Food Science, and Nutrition and Food Safety Research Institute, Universitat de Barcelona (UB), 08028 Barcelona, Spain; lluisa.miro@ub.edu (L.M.); camat@ub.edu (C.A.); cristina.rosell@ub.edu (C.R.-C.); mmoreto@ub.edu (M.M.); 2 APC-Europe S.L.U., 08403 Granollers, Spain; javier.polo@apc-europe.com; 3 APC LLC, 2425 SE Oak Tree Court, Ankeny, IA 50021, USA; Joy.Campbell@apcproteins.com

**Keywords:** spray-dried plasma, anti-inflammatory, colitis, barrier function

## Abstract

Dietary supplementation with spray-dried porcine plasma (SDP) can modulate the immune response of gut-associated lymphoid tissue. SDP supplementation reduces acute mucosal inflammation, as well as chronic inflammation associated with aging. The aim of this study was to analyze if SDP supplementation could ameliorate colitis in a genetic mouse model of inflammatory bowel disease (IBD). Wild-type mice and *Mdr1a* knockout (KO) mice were administered a control diet or an SDP-supplemented diet from day 21 (weaning) until day 56. The histopathological index, epithelial barrier, and intestinal immune system were analyzed in the colonic mucosa. KO mice had higher epithelial permeability, increased *Muc1* and *Muc4* expression, and lower abundance of E-cadherin and *Muc2* (all *p* < 0.001). SDP prevented these effects (all *p* < 0.05) and decreased the colonic inflammation observed in KO mice, reducing neutrophil and monocyte infiltration and activation and the percentage of activated T helper lymphocytes in the colonic mucosa (all *p* < 0.05). SDP also diminished proinflammatory cytokine expression and increased the anti-inflammatory IL-10 concentration in the colonic mucosa (all *p* < 0.05). In conclusion, dietary supplementation with SDP enhances colon barrier function and reduces mucosal inflammation in a mouse model of IBD.

## 1. Introduction

Inflammatory bowel disease (IBD) is a chronic inflammatory disorder of the gastrointestinal tract that is classically divided into Crohn disease and ulcerative colitis. In IBD, which is characterized by alternative recurrence and alleviation periods, patients suffer from abdominal pain, diarrhea, bloody stools, and weight loss [1]. It is a global disease, with increasing incidence and prevalence around the world [2].

The etiopathogenesis of IBD has not yet been elucidated, but environmental, gut microbial, and genetic factors all play important roles [3]. The intestinal inflammatory response in patients with IBD also involves deregulation of gut-associated lymphoid tissue (GALT) [4]. This tissue is divided into inductive sites, which include the mesenteric lymph nodes, Peyer’s patches (in the small intestine), colonic patches, and isolated lymphoid follicles, and into effector sites, which include intraepithelial lymphocytes and lymphocytes from the *lamina propria* [5]. Dysfunction of the innate and adaptive immune responses triggers the release of numerous proinflammatory cytokines, such as tumor necrosis factor-alpha (TNF-α) and interferon-gamma (IFN-γ), which results in epithelial cell damage and apoptosis and alteration of the epithelial barrier [4]. The epithelial barrier comprises epithelial cells (enterocytes), tight junction proteins, and the mucosal layer [6] and constantly interacts with the underlying immune cells of the GALT [7]. In IBD patients, the integrity of the epithelial barrier is compromised, as shown by increased paracellular permeability and lower levels of tight junction proteins [8].

Although some drugs are able to modify the course of the disease and maintain remission, they are not effective in all patients and their long-term use may have adverse effects [9]. Therefore, alternative treatment strategies for IBD are necessary. Nutritional interventions may be a good candidate. Using the *Mdr1a* knockout (KO) mouse model of colitis [10,11], we previously showed that serum-derived bovine immunoglobulins (SBI) reduce colon permeability and the expression of oxidative markers and proinflammatory cytokines in the colonic mucosa, as well as leukocyte infiltration in the lamina propria and mesenteric lymph nodes [12,13]. Moreover, SBIs improve mucositis symptoms, tissue damage scores, and neutrophil and lymphocyte percentages in a rat model of mucositis [14].

Another dietary supplement that is widely used in farm animals to enhance growth and reduce both morbidity and mortality is spray-dried porcine plasma (SDP) [15,16]. This supplement can modulate the intestinal immune response to inflammatory agents. For example, it reduces T lymphocyte activation, preventing the release of proinflammatory cytokines, and improves the mucosal barrier function after staphylococcal enterotoxin B challenge in rodents [17,18,19]. In senescent mice, SDP supplementation reduces nonspecific basal immune activation associated with age (inflammaging) by promoting mucosal regulatory T helper (Th) lymphocytes (Treg lymphocytes) and interleukin (IL)-10 production [18]. Moreover, SDP attenuates cognitive decline and reduces brain capillary permeability, oxidative stress, and proinflammatory cytokine expression in the brain of senescent mice [20].

Given the ability of SBI effects reducing the severity of colitis in the *Mdr1a* KO mouse model [12,13], we wanted to study if SDP, a compound with well-documented antioxidant and anti-inflammatory activities, can also modulate the immune response and ameliorate colitis in this animal model.

## 2. Results

### 2.1. Morphological Effects of SDP in Mdr1a KO Mice

**Figure 1:**
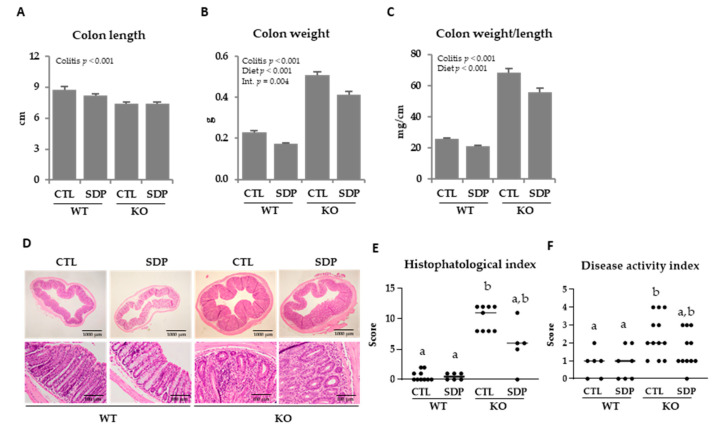
Effects of spray-dried porcine plasma (SDP) supplementation on colon morphology in *Mdr1a* knockout (KO) mice. (**A**) Colon length. (**B**) Colon weight. (**C**) Ratio between colon weight and colon length. (**D**) Representative images of hematoxylin and eosin staining from a histopathological study of the colonic mucosa. Top images were taken at low magnification (×2), whereas bottom images were taken at high magnification (×20). (**E**) Histopathological index. (**F**) Disease activity index. In panels A to C, results are expressed as mean ± SEM (*n* = 6–9 mice/group). In panels D and E, results are expressed as median (*n* = 5–12 mice/group). Means without a common letter differ, *p* < 0.05. CTL, control; Int, indicates the interaction between colitis and diet.

Colon of *Mdr1a* knockout (KO) mice were shorter and thicker than those of wild-type (WT) animals (*p* < 0.001; Figure 1A–C). The differences in colon diameter and wall thickness are clearly evident in the colon sections shown in Figure 1D. The SDP supplementation reduced colon weight and the ratio between colon weight and length (both, *p* < 0.001) while had no effects on colon length.

Histopathological and disease activity indices were increased in colitic mice (both *p* < 0.001; Figure 1E,F, respectively) and SDP supplementation had no significant effects. With the exception of ulceration, all specific parameters analyzed in the morphological study—mucus depletion, cryptitis, and lamina propria and transmural inflammation—were augmented in KO mice (all *p* < 0.05), and SDP did not modify these effects.

### 2.2. Effects of SDP on Colon Permeability in Mdr1a Knockout Mice

**Figure 2:**
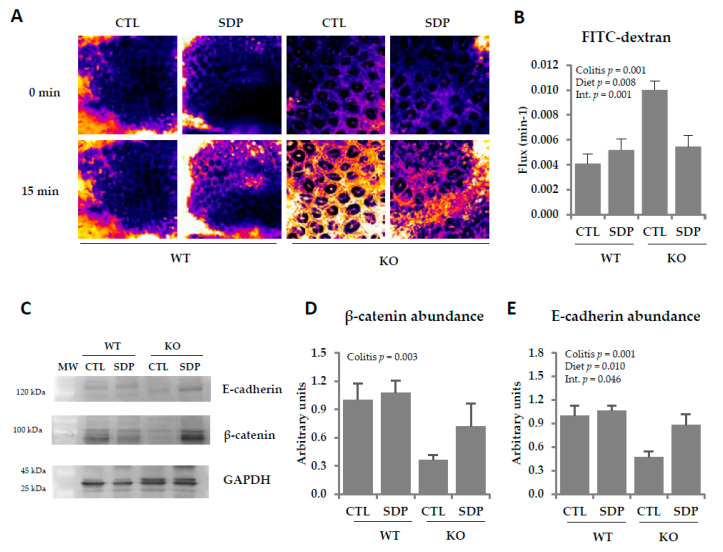
Effects of SDP supplementation on crypt permeability in the colonic mucosa of *Mdr1a* KO mice. (**A**) Representative images of FITC-dextran accumulation in the pericryptal sheath of the colonic mucosa after 15 min. All images were taken at the same magnification (×63). (**B**) Quantification of FITC-dextran accumulation in the pericryptal sheath of the colonic mucosa. (**C**) Representative Western blots of β-catenin (92 kDa), E-cadherin (132 kDa), and GAPDH (36 kDa). (**D**) β-catenin abundance in the colonic mucosa. (**E**) E-cadherin abundance in the colonic mucosa. Results are expressed as mean ± SEM (*n* = 5–7 mice/group). CTL, control; Int., indicates the interaction between colitis and diet; MW, molecular weight; SDP, spray-dried porcine plasma.

We next analyzed crypt permeability using 4KDa fluorescein isothiocyanate (FITC)-dextran accumulation (Figure 2A). The KO mice showed increased crypt permeability in the colon (*p* = 0.001; Figure 2B), with SDP supplementation preventing this increase (*p* = 0.001). Moreover, KO mice exhibited lower levels of β-catenin (*p* = 0.003, Figure 2C,D) and E-cadherin (*p* = 0.001, Figure 2C,E). E-cadherin abundance has been modified by SDP supplementation (*p* = 0.010), preventing the reduction observed in KO mice (*p* = 0.046).

**Figure 3:**
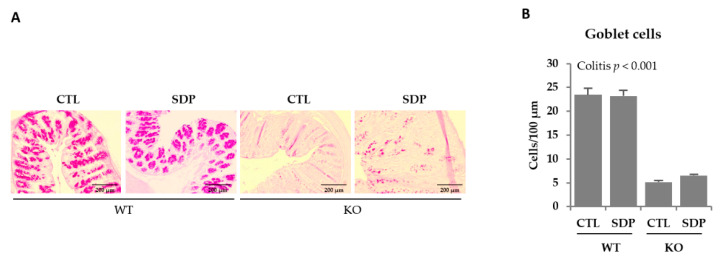
Effects of SDP supplementation on the levels of goblet cells in the colonic mucosa of *Mdr1a* KO mice. (**A**) Representative images periodic acid-Schiff staining of goblet cells in colon tissue. Images were taken at ×10 magnification. (**B**) Quantification of goblet cells in the colonic mucosa. Results are expressed as mean ± SEM (*n* = 5–7 mice/group).

KO mice showed increased expression of the transmembrane mucin 1 (*Muc1)* and mucin 4 (*Muc4*; both *p* < 0.001, Table 1). SDP supplementation reduced *Muc1* expression in KO mice (*p* = 0.016) and diminished *Muc4* expression in both WT and KO mice (*p* = 0.005). KO mice showed a substantial decrease in the expression of the secretory mucin 2 (*Muc2*; *p* < 0.001) and SDP supplementation attenuated this reduction (*p* = 0.023). Mucin 6 (*Muc6*) expression was similar in the two mouse groups and was also not altered by SDP supplementation. The KO animals had a lower expression of trefoil factor 3 (*Tff3*) than WT mice (*p* = 0.036) and SDP supplementation attenuated this effect, resulting in similar expression to that seen in WT mice (Interaction *p* = 0.053). Goblet cell number was examined using periodic acid-Schiff staining (Figure 3A). KO mice also had a lower number of goblet cells (*p* < 0.001, Figure 3B).

### 2.3. Effects of SDP on the Immune System in the Lamina Propria and Intraepithelial Lymphocytes in Mdr1a KO Mice

KO mice showed considerable immune cell recruitment in the lamina propria (*p* < 0.001, Table 2). The SDP supplementation reduced the immune cell infiltration (*p* = 0.003), but this reduction was higher in KO mice (*p* = 0.011). The levels of neutrophils and monocytes, as well as the percentages of activated neutrophils and monocytes, were higher in KO than WT mice (all *p* < 0.001) and SDP reduced these effects (*p* = 0.034; *p* = 0.044, *p* = 0.024, and *p* = 0.002, respectively). KO mice had a lower percentage of dendritic cells (*p* < 0.001) but the percentage of activated cells tended to be higher (*p* = 0.063). On the other hand, dietary supplementation with SDP enhanced the abundance of this subset of cells, as well as their activation in the lamina propria, in both mouse groups (both *p* < 0.001). In KO mice, there was an increase in the percentage of activated Th lymphocytes (*p* < 0.001) and SDP supplementation reduced this percentage in both animal groups (*p* = 0.009). On the other hand, although the percentage of Treg lymphocytes was augmented in KO mice (*p* < 0.001), SDP increased this percentage more in KO mice than in WT mice (*p* = 0.022), resulting in a lower ratio between activated Th and Treg lymphocytes (*p* < 0.001).

KO mice had more infiltrated immune cells in the intraepithelial compartment than WT mice, and the levels of monocytes and neutrophils were increased (all *p* < 0.001, Table 3). Dietary supplementation reduced the amount of infiltrated immune cells and the percentage of neutrophils (both *p* < 0.001). In addition, KO mice had higher percentages of activated monocytes and activated neutrophils, and both subsets were reduced by SDP supplementation (*p* = 0.007 and *p* = 0.030, respectively). Dendritic cells and their activation were reduced in KO mice (*p* = 0.025 and *p* = 0.003, respectively), and SDP supplementation did not modify their percentage. Regarding activated Th and Treg lymphocytes, the former were increased in KO mice (*p* < 0.001) and were unchanged by SDP supplementation. In contrast, the abundance of the latter was not different between WT and KO mice but was increased by SDP supplementation (*p* < 0.001). Consequently, KO animals showed a higher ratio of activated Th to Treg lymphocytes than animals supplemented with SDP (both *p* < 0.001).

### 2.4. Effects of SDP Suplementation on Inflammation Markers in Mdr1a KO Mice

**Figure 4:**
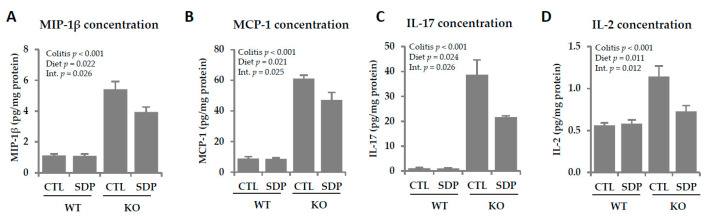
Effects of SDP supplementation on cytokine secretion in the colonic mucosa of *Mdr1a* KO mice. (**A**) MIP-1β concentration; (**B**) MCP-1 concentration; (**C**) IL-17 concentration; (**D**) IL-2 concentration. Results are expressed as mean ± SEM (*n* = 5–7 mice/group). CTL, control; IL-2, interleukin-2; IL-17, interleukin-17; Int., interaction between colitis and dietary factors; MCP-1, monocyte chemoattractant protein 1; MIP-1β, macrophage inflammatory protein 1 beta; SDP, spray-dried porcine plasma.

The concentrations of the chemokines MIP-1β and MCP-1 were augmented in the colonic mucosa in KO mice (both *p* < 0.001, Figure 4A,B) and SDP supplementation reduced their levels (*p* = 0.026 and *p* = 0.025, respectively). The concentrations of the cytokines IL-17 and Il-2 were also increased in KO mice (*p* < 0.001, Figure 4C,D), with SDP supplementation once again blocking the increase (*p* = 0.026 and *p* = 0.012, respectively).

**Figure 5:**
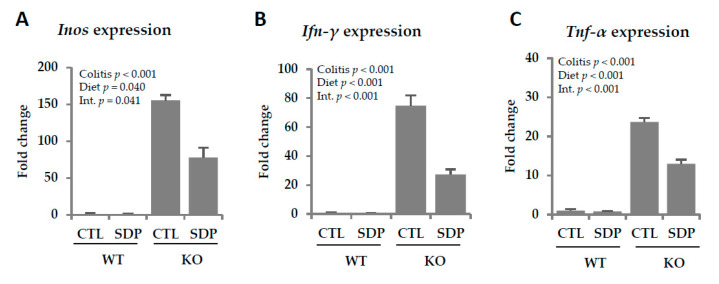
Effects of SDP supplementation on proinflammatory cytokine expression in the colonic mucosa of *Mdr1a* KO mice. (**A**) Inos expression; (**B**) Ifn-γ expression; (**C**) Tnf-α expression. Results are expressed as mean ± SEM (*n* = 4–7 mice/group). CTL, control; Inos, inducible nitric oxide synthase; Ifn-γ, interferon-gamma; Int., indicates the interaction between colitis and dietary factors; SDP, spray-dried porcine plasma; Tnf-α, tumor necrosis factor-alpha.

In KO mice, the expression levels of *Inos*, *Ifn-γ*, and *Tnf-α* were increased (all *p* < 0.001, Figure 5), and SDP supplementation reduced all these effects (*p* = 0.041, *p* < 0.001, and *p* < 0.001, respectively).

**Figure 6:**
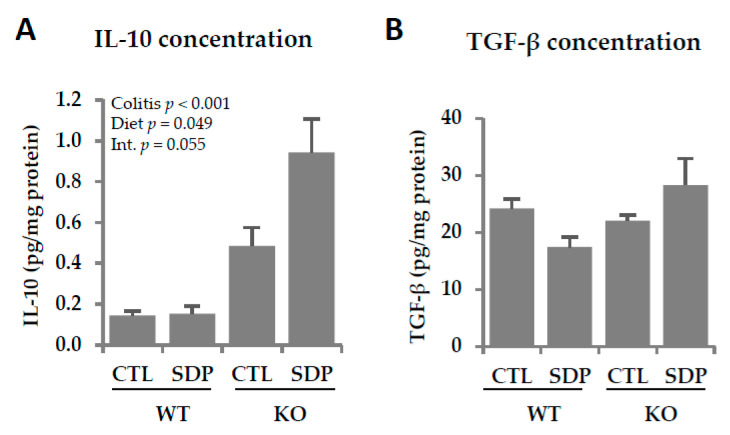
Effects of SDP supplementation on anti-inflammatory cytokine secretion in the colonic mucosa of *Mdr1a* KO mice. (**A**) IL-10 concentration; (**B**) TGF-β concentration. Results are expressed as mean ± SEM (*n* = 5–7 mice/group). CTL, control; IL-10, interleukin-10; Int., interaction between colitis and dietary factors; SDP, spray-dried porcine plasma; TGF-β, transforming growth factor-beta.

SDP supplementation increased the concentration of the anti-inflammatory cytokine IL-10 (*p* = 0.049, Figure 6A). No changes in the TGF-β concentration were observed in the colonic mucosa in KO mice or after SDP supplementation (Figure 6B).

## 3. Discussion

The pathogenesis of IBD is still unknown. However, it is well-established that patients have a deregulated immune response involving increases in proinflammatory cytokines, which has been identified to contribute to disorders of the gastrointestinal tract [21]. In recent years, nutraceutical compounds, which include bioactive peptides and colostrum, have been reported to have beneficial effects in IBD patients [22]. Therefore, in the present study, we evaluated the effects of dietary supplementation with SDP on the evolution of IBD in a mouse model of spontaneous colitis.

The gastrointestinal tract can develop different inflammatory pathologies, with changes in epithelial permeability leading to altered mucosal functions [23]. Here, *Mdr1a* KO mice showed an increase in the histopathological index, indicating intestinal deterioration. In addition, KO mice had higher epithelial permeability and lower abundance of tight junction proteins than WT animals, which is consistent with the alterations in epithelial permeability and in tight junction structure observed in patients with ulcerative colitis and Crohn disease [8]. Although SDP supplementation did not improve the histopathological index, it prevented the increase in crypt epithelial permeability and the reduction in E-cadherin abundance observed in *Mdr1a* KO animals. This effect is similar to that observed in the same model of colitis after supplementation with SBI, which is an immunoglobulin concentrate [12]. Previous work has also shown that SDP can prevent increases in small intestine permeability during acute intestinal inflammation induced by an enterotoxin [17].

IBD patients show lower abundance of goblet cells and changes in mucin expression and secretion [24], which reduce the thickness of the mucus layer [25] and thereby compromise barrier function. In addition to mucins, goblet cells also synthesize trefoil factors, which are secretory proteins that stabilize the mucus layer [26] and facilitate intestinal epithelial restitution [27]. Our *Mdr1a* KO mice showed a reduction in the number of goblet cells in the colon, as well as in the expression of *Muc2* and *Tff3*, which would contribute to an increase in the epithelial permeability of the colon [28]. Indeed, it has been described that a reduction in goblet cells and *Muc2* would be associated with an increase in pathogenic bacteria in the mucus layer and a greater penetration of these microorganisms into the mucosa [29]. SDP supplementation did not improve goblet cell abundance in KO mice but did attenuate the decrease in *Muc2* expression and maintained *Tff3* expression at similar levels to those of healthy animals. Therefore, SDP helps to maintain barrier integrity and prevent the entry of pathogenic microorganisms that could eventually perpetuate inflammation in the colonic mucosa.

Both MUC1 and MUC4 are membrane-bound mucins that are elevated in patients with IBD during the inactive phase of the disease, suggesting a crucial role of these mucins in the development of the condition [30]. In addition, aberrant expression of *Muc1* is involved in intestinal barrier dysfunction during inflammation, with overexpression of *Muc1* correlated with increased intestinal permeability [31]. On the other hand, neutrophil infiltration is associated with increased *Muc4* expression [32]. SDP supplementation reduced the overexpression of *Muc1* and *Muc4* observed in KO mice, suggesting that SDP-supplemented mice had lower immune activity and an enhanced colon barrier.

*Mdr1a* KO mice showed an increase in immune cell recruitment in the *lamina propria* and in the intraepithelial compartment, as observed in IBD patients [33]. This infiltration is triggered by monocytes, neutrophils, and Th lymphocytes [34]. The infiltration is accompanied by chemokines and cytokines that promote the recruitment and activation of these cells, leading to unrestrained accumulation of activated immune cells that impair mucosal homeostasis and perpetuate the inflammatory response [35]. We found that SDP supplementation reduced the accumulation of activated monocytes and neutrophils in the colonic mucosa, which is consistent with reduced production of MIP-1β and MCP-1. This effect is notable because these chemokines are important mediators for the recruitment and accumulation of neutrophils and macrophages in colitis models [36]. The SDP diet also reduced the percentage of activated Th cells in the *lamina propria*, diminishing an immune subset that plays a main role in initiating and shaping IBD pathogenesis [37]. The decline in Th cell activation led to a reduction in proinflammatory cytokines, such as IL-2 and IFN-γ. This is noteworthy because activated Th lymphocytes are also characterized by alterations in cytokine production, which result in a disturbed balance between pro- and anti-inflammatory cytokines [38,39]. Along this line, similar effects of SDP supplementation have been observed in other rodent models of jejunum and pulmonary acute inflammation [40,41,42].

Th17 cells play an important role in host defense against extracellular pathogens but are also associated with the development of inflammatory responses such as those seen in IBD [43]. Here, *Mdr1a* KO mice showed a considerable increase in IL-17 release in the colonic mucosa, indicating a clear bias toward the Th17 immune response. SDP decreased the concentration of IL-17 in the colonic mucosa. This effect may be clinically relevant because IL-17 induces the release of chemokines and other chemoattractants from epithelial and endothelial cells that promote the inflammatory response through the recruitment of neutrophils [44]. Therefore, the reduced IL-17 release observed in KO mice supplemented with SDP might explain the lower activation of neutrophils and their reduced infiltration into the colonic mucosa. Similar effects of SDP supplementation were observed in mice with acute lung inflammation induced by lipopolysaccharide (LPS), with a reduction in the activation of neutrophils and their infiltration into lung tissue [42,45].

Immune activation and proinflammatory cytokine release can disassemble tight junction proteins, increasing epithelial permeability [46]. This can trigger and perpetuate local inflammation in IBD [47]. Furthermore, both *Tnf-α* and *Ifn-γ* induce *Inos* expression, which also has deleterious effects on intestinal integrity [48]. In general, our results showed that SDP supplementation reduced the immune response during colitis development. A similar pattern was also observed with SBI supplementation in *Mdr1a* KO mice [13].

The intestinal mucosa is normally maintained in a state of controlled inflammation in which there is a balance between protective immune responses and tolerance to self-antigen and commensal bacteria [49]. Dendritic cells participate in the preservation of Th intestinal tolerance through the activation and maintenance of Treg cells [50], which in turn control immune responses in the gut by inhibiting the proliferation and effector functions of other T cells. Inflammation in IBD may be caused by an alteration in the balance between Treg and proinflammatory activated Th cells [51]. Our KO mice showed a reduced proportion of dendritic cells and activated dendritic cells, which correlates well with the reduction in the Treg population. In our experiments, SDP supplementation increased the percentage of dendritic cells and their activation in the lamina propria of the KO mice, as well as the proportion of Treg lymphocytes in the colonic mucosa. SDP supplementation also reduced the ratio between activated Th lymphocytes and Treg lymphocytes, indicating that SDP restores the balance between these lymphocyte populations. A similar response pattern has been observed in the Staphylococcal enterotoxin B model of mild intestinal inflammation [19] and in acute lung inflammation induced by LPS [45]. In both cases, the challenge increased the activated Th/Treg ratio while SDP restored the pre-challenge ratio. The importance of these effects lies in the role of Treg in the suppression of Th effector cells through the secretion of anti-inflammatory cytokines such as IL-10 and TGF-β [52].

SDP supplementation did not modify colon TGF-β concentration but did promote IL-10 release. The lack of a TGF-β response was unexpected because, in other inflammation models, such as intestinal and genitourinary acute inflammation models, SDP induces the secretion of TGF-β [41,53]. However, in acute lung inflammation, only mature TGF-β (and not total TGF-β) is increased by SDP [42].

On the other hand, SDP supplementation increased the release of IL-10 during chronic inflammation. These results are consistent with the acute intestinal inflammation induced by staphylococcal enterotoxin B [19], with the acute lung inflammation provoked by LPS [42], and with the uterine inflammation induced by stress [53], in which SDP supplementation increases the expression of this anti-inflammatory cytokine. In a very different condition, such as in a mouse model of senescence, SDP increases the brain concentration of IL-10, suggesting its participation in the regulation of immune responses in nonmucosal tissues [20]. All of these effects of SDP on IL-10 are remarkable because this cytokine plays an important anti-inflammatory role in restricting and suppressing the inflammatory responses, thus minimizing tissue damage in response to microbial challenges [54].

Besides the effect of SDP on the immune system itself, it can also act through changes in the intestinal microbiota. In this regard, Moretó et al. [55] showed that supplementation with SDP increases the proportion of bacterial families that improve intestinal barrier function and are well-known mediators of anti-inflammatory and tolerogenic responses, such as the Lactobacillaceae family. In fact, different species of the genus *Lactobacillus* have been shown to be effective in promoting the expression of *Il-10* [22] or in reducing the expression of adhesion molecules that promote leukocyte recruitment in an experimental colitis model [56].

## 4. Materials and Methods

### 4.1. Animals and Experimental Design

Mdr1a knockout mice (FVB.129P2-Abcb1atm 1BorN7; KO mice) and the corresponding FVB wild type (WT mice) were purchased from Taconics (Germtown, NY, USA). We created a stable colony for KO animals in the specific pathogen free (SPF) area of the Animal Experimentation Service of the Barcelona Science Park (BSP). Animals were kept under stable temperature and humidity conditions, with a 12 h:12 h light/dark cycle. All protocols used in this study were approved by the Ethics Committee for Animal Experimentation of the BSP (Permit number: P03-R1-08, approved 09/05/2008).

The animals were weaned at day 21 of age and then consumed the experimental diets until the end of the study. The animals were fed a control diet, or a diet supplemented with 8% of SDP, which is a protein-rich ingredient obtained from industrial fractionation of blood from healthy pigs intended for human consumption. Control and SDP diets were designed to provide balanced energy and nutrients. The experimental diets were prepared by APC-Europe S.L.U (Granollers, Spain) from base ingredients provided by Envigo (Bresso, Italy), and their composition is detailed in Table 4. The experimental groups used throughout the study were WT (WT mice fed control diet), KO (KO mice fed control diet), SDP (WT mice fed SDP diet), and KO-SDP (KO mice fed the SDP diet). Mice were maintained in the SPF area until day 28 of age, when they were transferred to a conventional housing area. Animals were killed by anesthesia overdose at 56 days of age.

The clinical signs of colitis were analyzed using the disease activity index (DAI), which scores weight loss, stool consistency and bleeding, as described elsewhere [12].

### 4.2. Morphological Study

This procedure was carried out as described before [13]. Histopathological analysis was performed by the Pathological Anatomy Department of the Vall d’Hebron Hospital (Barcelona, Spain), under blind conditions. Analysis of the degree of histological alterations assigned values between 0–3 (absent-slight-moderate-severe). The variables studied were lamina propria and transmural cellular infiltration, cryptitis, ulcers and mucus depletion.

### 4.3. Intraepithelial Lymphocytes and Lamina Propria Lymphocytes Isolation

Lymphocytes isolation was carried out as previously described [13,57]. Briefly, the colon was extracted, washed with PBS and everted. Then, the tissue was incubated in a pre-digestion solution HBSS with 10% FBS (both Invitrogen, Carlsbad, CA, USA), 5 mM EDTA and 1 mM DTT (both Sigma-Aldrich, St. Louis, MO, USA) for 25 min at 37 °C in a shaker (Thermomixer Comfort Eppendorf^®^, 750 rpm). The cell suspension was filtered in a cell strainer of 75 μm and centrifuged at 500× *g* for 10 min at 4 °C. The pelleted cells were resuspended in PBS-FBS and reserved until do the Percoll gradient. The cell suspension contains intraepithelial lymphocytes among other cell types.

The remainder colon was finely minced and incubated in digestion solution containing RPMI 1640, 5% FBS, penicillin/streptomycin, 10 nM HEPES, 2 mM L-Glutamine and 1500 U/mL collagenase (all, Invitrogen, Carlsbad, CA, USA) at 37 °C for 20 min in a shaker (Thermomixer Comfort Eppendorf^®^, 750 rpm). The cell suspension obtained was separated and the rest of tissue was incubated two additional times with the digestion solution. The cell suspension obtained was filtered in a 75 μm cell strainer and centrifuged at 500× *g* for 10 min at 4 °C. The pelleted cells were resuspended in PBS-FBS. This cell suspension contains the lamina propria lymphocytes among others cell types.

At this point, both types of enriched cell suspensions (from lamina propria and intraepithelial compartment) were proceed at the same time. Samples were centrifuged at 500× *g* for 10 min at 4 °C and were submit to gradient Percoll 40–80%. Leukocytes were transferred to a clean tube and centrifuged at 500× *g* for 10 min at 4 °C. The pelleted cells were resuspended in PBS-FBS. Cell number and viability were determined using acridine orange and ethidium bromide markers. In all cases, cell viability was >90%.

### 4.4. Cell Staining

Cell staining was performed as described before [13]. Briefly, the staining was carried out on 1.5 × 10^4^ cells in samples of the lamina propria, and on 7 × 10^4^ cells of the intraepithelial subset. To stain extracellular markers, cells were incubated with the primary antibodies CD45, CD19, CD4, CD25, CD14 and CD11c (all from eBioscience Inc., San Diego, CA, USA) and LY6G and CD86 (BD Pharmingen, Franklin Lakes, NJ, USA) for 30 min at 4 °C. To stain the intracellular markers, cells were fixed with paraformaldehyde 4% for 20 min at 4 °C (Sigma-Aldrich, St. Louis, MO, USA), and permeabilized with Triton-X^®^ for 15 min at 4 °C (Sigma-Aldrich, St. Louis, MO, USA). Cells were incubated with the primary antibodies FOXP3 (eBioscience Inc. San Diego, CA, USA) and CD68 (Serotec GmbH Kidlington, UK) for 30 min at 4 °C. Finally, cells were maintained in paraformaldehyde 4% until further analysis in the Aria cell sorter (Becton Dickinson, Franklin Lakes, NJ, USA) located at the Cytometry Unit of the Technical Services of the Universitat de Barcelona at the BSP. Results were analyzed using the Flowjo Software (Treestar Inc., Ashland, OR, USA). Cell markers to quantify each leukocyte population are detailed in Table 5.

### 4.5. Epithelial Fluorescent Dextran Permeability

This protocol was performed as previously described [12]. Briefly, the colon mucosa was scrapped and kept for 15 min in a petri dish which contained carbogenated Earl’s solution at 37 °C to stabilize the sample. The fluorescent tracer fluorescein isothiocyanate (FITC)-labeled dextran (FITC-dextran; molecular weight 4000; Sigma-Aldrich, St. Louis, MO, USA) was used to study the crypt permeability. To carry out the monitoring of the ratio of fluorescence intensity of crypt luminal and pericryptal areas, tissue samples were incubated for 15 min and confocal images were taken every 3 min with a CLSM SPII confocal microscope (Leica Microsystems, Heidelberg, Germany). Two fields were captured per tissue per animal. The image was quantified with ImageJ software.

### 4.6. Western Blot

This procedure was carried out as described before [55]. Samples of colon mucosa were homogenized. Equal amounts of protein (100 µg) were separated on 10% SDS-PAGE and transferred to polyvinylidene difluoride membranes (Bio-Rad, Hercules, CA, USA). Membranes were incubated overnight at 4 °C with specific primary antibodies against GAPDH (clone GAPDH-71.1; 1:10,000; Sigma-Aldrich, St. Louis, MO, USA), β-catenin (1:2000; NeoMarkers, Fremont, CA, USA), E-cadherin (clone H-108, 1:500; Santa Cruz Biotechnology, Dallas, TX, USA). The blots were developed by using WesternDot 625 Western blot kits (Life Technologies, Paisley, UK) and quantified with ImageJ gel analyser software.

### 4.7. Real-Time PCR

RNA isolation, reverse transcription was performed as describe previously [20]. Total RNA was retrotranscribed using an iScript cDNA Synthesis Kit (Bio-Rad, Hercules, CA, USA). Real-time PCR was performed using a cDNA template in a 20 μL reaction containing 0.2 μmol/L of each primer and SsoAdvanced Universal SYBR Green Supermix (Bio-Rad, Hercules, CA, USA) and carried out on a MiniOpticon Real-Time PCR System (Bio-Rad, Hercules, CA, USA). The primers used to detect the genes of interest were cited elsewhere [18,45]. The target gene transcripts were quantified using glyceraldehyde-3-phosphate dehydrogenase (GAPDH) gene expression as a reference, using the 2^−∆∆*C*t^ method [58]. Product fidelity was confirmed by melt-curve analysis.

### 4.8. Cytokine Concentration

Samples of colon mucosa were homogenized as described before [13]. Cytokines were measured by Bio-Plex Cytokine Assay™ (Bio-Rad, Hercules, CA, USA).

### 4.9. Statistical Analysis

Results are presented as means ± SEM except for the disease activity index score, histopathological index and parameters used in the histopathological index, which are expressed as medians. A test was performed to detect outliers (Grubb test) and check the homogeneity of variance (Shapiro-Wilk test) and data normality (Shapiro–Wilk test) for all groups. Median values for nonparametric data were compared by the Kruskal-Wallis test. The effects of diet, colitis as well as their interaction (diet and colitis factors) were analyzed by two-way ANOVA followed by Fisher’s Least Significant Difference (LSD) post hoc test (applied between the different diets), using GraphPad Prism^®^ software v 7.01 (GraphPad Software, Inc., La Jolla, CA, USA). Statistical differences were considered significant at *p* < 0.05. A *p* value between 0.05 and 0.1 was suggestive of a true effect [59].

## 5. Conclusions

In conclusion, our findings showed that dietary supplementation with SDP reduces the amount of activated Th lymphocytes and promotes the presence of Treg cells in mice that spontaneously develop colitis; in turn, SDP increases the secretion of Il-10, which favored an anti-inflammatory immune profile. This cascade eventually reduces proinflammatory cytokines in the mucosa and restores the barrier properties of the colonic epithelium. The beneficial effect of SDP in this model suggests a role for plasma-derived supplements in preventing and ameliorating IBD.

## Figures and Tables

**Table 1:** Effects of SDP Supplementation on The Expression of *Mucin 1* (*Muc1*), *Mucin 2* (*Muc2*), *Mucin 4* (*Muc4*), *Mucin 6* (*Muc6*), and trefoil factor 3 (*Tff3*) in The Colonic Mucosa of *Mdr1a* knockout (KO) Mice.

	WT		KO		*p* Value		
	CTL	SDP	CTL	SDP	Colitis	Diet	Int.
*Muc1*	1.00 ± 0.04	1.98 ± 0.03	5.90 ± 0.50	5.00 ± 0.36	<0.001	ns	0.016
*Muc2*	1.00 ± 0.09	0.84 ± 0.04	0.21 ± 0.03	0.35 ± 0.03	<0.001	ns	0.023
*Muc4*	1.00 ± 0.17	0.59 ± 0.02	2.86 ± 0.31	1.85 ± 0.10	<0.001	0.005	ns
*Muc6*	1.00 ± 0.20	0.96 ± 0.36	1.17 ± 0.17	0.90 ± 0.15	ns	ns	ns
*Tff3*	1.00 ± 0.16	0.97 ± 0.09	0.42 ± 0.08	0.92 ± 0.13	0.036	ns	0.053

Results are expressed as mean ± SEM (*n* = 3–5 mice/group). CTL, control; Int., interaction between colitis and dietary factors; Muc1, mucin 1; Muc2, mucin 2; Muc4, mucin 4; Muc6, mucin 6; ns, nonsignificant; SDP, spray-dried porcine plasma; Tff3, trefoil factor 3.

**Table 2:** Effects of SDP Supplementation on The Immune Populations of The Lamina Propria of The Colonic Mucosa of *Mdr1a* KO Mice.

	WT		KO		*p* Value		
	CTL	SDP	CTL	SDP	Colitis	Diet	Int.
Cell count (×10^6^)	1.55 ± 0.17	1.14 ± 0.19	16.0 ± 1.07	11.1 ± 0.97	<0.001	0.003	0.011
Monocytes	8.10 ± 0.58	7.74 ± 0.79	13.0 ± 0.49	10.5 ± 0.83	<0.001	0.044	ns
Act. monocytes	29.8 ± 2.72	27.1 ± 1.74	60.3 ± 1.70	49.3 ± 1.89	<0.001	0.002	ns
Neutrophils	19.0 ± 2.19	17.5 ± 2.52	38.9 ± 1.39	31.4 ± 2.08	<0.001	0.034	ns
Act. neutrophils	31.1 ± 2.78	25.7 ± 2.30	57.8 ± 2.21	51.5 ± 2.53	<0.001	0.024	ns
Dendritic cells	19.7 ± 1.50	23.7 ± 1.38	9.49 ± 0.41	12.2 ± 0.60	<0.001	<0.001	ns
Act. dendritic cells	4.51 ± 0.79	9.29 ± 1.29	6.36 ± 0.46	10.7 ± 1.34	0.063	<0.001	ns
Act. Th lymphocytes	6.80 ± 0.69	5.32 ± 0.58	17.0 ± 0.85	13.8 ± 0.60	<0.001	0.009	ns
Treg lymphocytes	3.26 ± 0.45	3.34 ± 0.53	5.77 ± 0.52	8.81 ± 0.55	<0.001	0.017	0.022
Act. Th/Treg lym. ratio	2.51 ± 0.22	1.66 ± 0.19	3.09 ± 0.27	1.60 ± 0.09	ns	<0.001	0.040

Results are expressed as mean ± SEM (*n* = 5–14 mice/group). Cell values are expressed as absolute number for cell count while monocytes, neutrophils, and dendritic cells are expressed as the percentage of nonlymphocytic leukocytes. Activated and regulatory cells are expressed as the percentage of the corresponding total subset. Act., activated; CTL, control; Int., interaction between colitis and dietary factors; ns, nonsignificant; SDP, spray-dried porcine plasma; Th, T helper; Treg; regulatory T helper.

**Table 3:** Effects of SDP Supplementation on The Immune Population of The Intraepithelial Compartment In The Colonic Mucosa of *Mdr1a* KO mice.

	WT		KO		*p* Value		
	CTL	SDP	CTL	SDP	Colitis	Diet	Int.
Cell count (×10^6^)	0.26 ± 0.06	0.19 ± 0.05	1.76 ± 0.22	0.81 ± 0.10	<0.001	<0.001	0.003
Monocytes	9.39 ± 0.60	8.21 ± 1.55	12.8 ± 0.78	11.1 ± 0.91	<0.001	ns	ns
Act. monocytes	5.90 ± 1.17	3.88 ± 0.49	19.6 ± 1.71	13.1 ± 0.58	<0.001	0.007	ns
Neutrophils	8.09 ± 0.75	5.61 ± 0.54	15.5 ± 0.94	9.82 ± 0.54	<0.001	<0.001	ns
Act. neutrophils	4.43 ± 1.13	6.31 ± 0.99	21.7 ± 1.83	12.7 ± 0.83	<0.001	0.030	0.002
Dendritic cells	20.1 ± 2.55	19.1 ± 2.22	15.0 ± 1.42	16.4 ± 0.76	0.025	ns	ns
Act. dendritic cells	20.9 ± 2.78	20.0 ± 2.02	13.8 ± 0.68	17.7 ± 0.63	0.003	ns	ns
Act. Th lymphocytes	6.08 ± 0.18	6.37 ± 0.76	16.1 ± 0.94	13.0 ± 0.74	<0.001	ns	ns
Treg lymphocytes	3.10 ± 0.43	5.33 ± 0.26	3.39 ± 0.21	5.27 ± 0.47	ns	<0.001	ns
Act. Th/Treg lym. ratio	2.09 ± 0.31	1.19 ± 0.09	4.83 ± 0.26	2.58 ± 0.18	<0.001	<0.001	0.015

Results are expressed as mean ± SEM (*n* = 5–14 mice/group). Cell values are expressed as absolute number for cell count while monocytes, neutrophils, and dendritic cells are expressed as the percentage of nonlymphocytic leukocytes. Activated and regulatory cells are expressed as the percentage of the corresponding total subset. Act, activated; CTL, control; Int., interaction between colitis and dietary factors; ns, nonsignificant; SDP, spray-dried porcine plasma; Th, T helper; Treg; regulatory T helper.

**Table 4:** Diet composition.

Ingredients	Control Diet	Ingredients
SDP ^1^	-	80
Dried skim milk	530.7	340.5
Corn starch	199.3	308.8
Sucrose	94.5	94.5
Soybean oil	70	70
Cellulose	50	50
AIN-93-G-MX ^2^	35	35
AIN-93 VX ^2^	15	15
Choline bitartrate	3	3
Methionine	2.5	3.2

^1^ SDP (spray-dried porcine plasma) was provided by APC-Europe S.L.U (Granollers, Spain); ^2^ AIN-93 VX, vitamin mix; AIN-93-G-MX, mineral mix, both provided by Envigo (Bresso, Italy).

**Table 5:** Cell Markers to Quantify Leukocyte Populations.

Population	Gate
Leukocytes	CD45^+^
B lymphocytes	CD19^+^CD45^+^
T helper cells	CD4^+^CD45^+^
Activated Th lymphocytes	CD25^+^FOXP3^−^CD4^+^
Regulatory Th lymphocytes	CD25^+^FOXP3^+^CD4^+^
Neutrophils	CD45^+^ non-lymphocytic leucocytes were separated by forward/side scatter Ly6G^+^
Monocytes	CD45^+^ non-lymphocytic leucocytes were separated by forward/side scatter CD68^+^
Activated neutrophils	CD45^+^ non-lymphocytic leucocytes were separated by forward/side scatter Ly6G^+^CD14^+^
Activated monocytes	CD45^+^ non-lymphocytic leucocytes were separated by forward/side scatter CD68^+^CD14^+^
Dendritic cells	CD11c^+^CD45^+^
Activated dendritic cells	CD45^+^CD11c^+^CD86^+^

## Abbreviations

CTL	Control
FITC	Fluorescein isothiocyanate
GALT	Gut-associated lymphoid tissue
IBD	Inflammatory bowel disease
IFN-γ	Interferon-gamma
IL	Interleukin
Inos	Inducible nitric oxide synthase
KO	Knockout
MCP-1	Monocyte chemoattractant protein 1
MIP-1β	Macrophage inflammatory protein 1 beta
Muc1	Mucin 1
Muc2	Mucin 2
Muc4	Mucin 4
Muc6	Mucin 6
MW	Molecular weight
SDP	Spray-dried porcine plasma
Tff3	Trefoil factor 3
TGF-β	Transforming growth factor-beta
Th	T helper
Treg	Regulatory Th lymphocytes
TNF-α	Tumor necrosis factor-alpha
WT	Wild type
